# 

**Published:** 1903-04

**Authors:** 


### CONTENTS.

ORIGINAL COMMUNICATIONS—

SOME THEORIES AS TO THE CAUSATION OF CANCER. By Greer Baughman, M. D., Richmond, Va. .......... 1
X-RAYS IN THE TREATMENT OF SUPERFICIAL CANCERS. By Alfred L. Gray, M. D., Richmond, Va. ........ 7
REPORT OF AN INTERESTING CASE. By O. L. Holmes, M. D., Stewart, Ga. ........ 11
ANKYLOSTOMIASIS DUODENALE. By A. B. McMillan, M. D., Burnt Corn, Ala. ........ 13
THE DIAGNOSIS OF CUTANEOUS CANCERS. By F. H. Beadles, M. D., Richmond, Va. ........ 16
THE TREATMENT OF CHRONIC BRONCHITIS. By Robert C. Kenner, M. D., Louisville, Ky. ........ 17
CORRESPONDENCE ........ 22
SOCIETY REPORTS ........ 24

EDITORIAL NOTES AND COMMENTS—

THE MAYBRICK CASE AGAIN ........ 42
THE POPULARIZATION OF MECHANICAL THERAPEUTICS ........ 42
INTRAVENOUS USES OF FORMALIN IN THE TREATMENT OF SEPTICEMIA ........ 44
FLOWER WITHIN THE CRANNIED WALLS ........ 44

CONTENTS CONTINUED ........ X

